# Particle beam therapy for pelvic recurrence of colorectal cancer: a registry data analysis in Japan and a systematic review

**DOI:** 10.1093/jrr/rrad024

**Published:** 2023-04-28

**Authors:** Shigeyuki Murayama, Shigeru Yamada, Yuichi Hiroshima, Hirotoshi Takiyama, Hiroshi Taguchi, Takuya Kimoto, Makoto Anzai, Yasuhito Hagiwara, Kazuaki Yasui, Keita Mori, Soichiro Ishihara, Hideki Ueno, Shinichi Shimizu, Hidefumi Aoyama, Hiroshi Tsuji, Hideyuki Sakurai

**Affiliations:** Division of Proton Therapy, Radiation and Proton Therapy Center, Shizuoka Cancer Center Hospital, 1007 Shimonagakubo, Nagaizumi-cho, Suntou-gun, Shizuoka 411-8777, Japan; The Japanese Society for Radiation Oncology, Particle Therapy Division, Colorectal Cancer Working Group; The Japanese Society for Radiation Oncology, Particle Therapy Division, Colorectal Cancer Working Group; QST Hospital, National Institutes for Quantum Science and Technology, 4-9-1 Anagawa, Inage-ku, Chiba-shi, Chiba 263-8555, Japan; The Japanese Society for Radiation Oncology, Particle Therapy Division, Colorectal Cancer Working Group; Department of Radiation Oncology & Proton Medical Research Center, Faculty of Medicine, University of Tsukuba, 1-1-1 Tennodai, Tsukuba, Ibaraki 305-8575, Japan; The Japanese Society for Radiation Oncology, Particle Therapy Division, Colorectal Cancer Working Group; QST Hospital, National Institutes for Quantum Science and Technology, 4-9-1 Anagawa, Inage-ku, Chiba-shi, Chiba 263-8555, Japan; The Japanese Society for Radiation Oncology, Particle Therapy Division, Colorectal Cancer Working Group; Department of Radiation Oncology, Hokkaido University Faculty of Medicine, Kita 15, Nishi 7, Kita-ku, Sapporo, Hokkaido 060-8638, Japan; The Japanese Society for Radiation Oncology, Particle Therapy Division, Colorectal Cancer Working Group; Department of Radiology, Kyoto Prefectural University of Medicine, 465 Kajiicho, Kamigyo-ku, Kyoto 602-0841, Japan; The Japanese Society for Radiation Oncology, Particle Therapy Division, Colorectal Cancer Working Group; Department of Radiology, Osaka Heavy Ion Therapy Center, 3-1-10 Otemae, Chuo-ku, Osaka-city, Osaka 540-0008, Japan; The Japanese Society for Radiation Oncology, Particle Therapy Division, Colorectal Cancer Working Group; Department of Radiology, Division of Radiation Oncology, Yamagata University Faculty of Medicine, 2-2-2, Iidanishi, Yamagata city, Yamagata 990-9585, Japan; Division of Radiation Oncology, Radiation and Proton Therapy Center, Shizuoka Cancer Center Hospital, 1007 Shimonagakubo, Nagaizumi-cho, Suntou-gun, Shizuoka 411-8777, Japan; Clinical Research Support Center, Shizuoka Cancer Center Hospital, 1007 Shimonagakubo, Nagaizumi-cho, Suntou-gun, Shizuoka 411-8777, Japan; Department of Surgical Oncology, The University of Tokyo, 7-3-1 Hongo, Bunkyo-ku, Tokyo 113-8655, Japan; Department of Surgery, National Defense Medical College, 3-2 Namiki, Tokorozawa, Saitama 359-8513, Japan; Department of Carbon Ion Radiotherapy, Graduate School of Medicine, Osaka University, 3-1-10, Otemae, Chuo-ku, Osaka City, Osaka 540-0008, Japan; Department of Radiation Oncology, Hokkaido University Faculty of Medicine, Kita 15, Nishi 7, Kita-ku, Sapporo, Hokkaido 060-8638, Japan; QST Hospital, National Institutes for Quantum Science and Technology, 4-9-1 Anagawa, Inage-ku, Chiba-shi, Chiba 263-8555, Japan; Department of Radiation Oncology & Proton Medical Research Center, Faculty of Medicine, University of Tsukuba, 1-1-1 Tennodai, Tsukuba, Ibaraki 305-8575, Japan

**Keywords:** colorectal cancer, pelvic recurrence, particle beam therapy, proton therapy, carbon ion radiation therapy, survival, toxicity

## Abstract

The aim of this study was to investigate the efficacy and safety of particle beam therapy (PBT) with proton or carbon ion beam for pelvic recurrence of colorectal cancer (PRCC) by comparing the clinical outcomes of a dataset of prospectively enrolled patients for PBT with those from the literature, which were collected by a systematic review of external X-ray radiotherapy (XRT) and PBT. Patients with PRCC treated at 14 domestic facilities between May 2016 and June 2019 and entered the database for prospective observational follow-up were analyzed. The registry data analyzed included 159 PRCC patients treated with PBT of whom 126 (79%) were treated with carbon ion radiation therapy (CIRT). The 3-year overall survival and local control rate were 81.8 and 76.4%, respectively. Among these PRCC patients, 5.7% had Grade 3 or higher toxicity. Systematic search of PubMed and Cochrane databases published from January 2000 to September 2020 resulted in 409 abstracts for the primary selection. Twelve studies fulfilled the inclusion criteria. With one additional publication, 13 studies were selected for qualitative analysis, including 9 on XRT and 4 on PBT. There were nine XRT studies, which included six on 3D conformal radiotherapy and three on stereotactic body radiation therapy, and four PBT studies included three on CIRT and one on proton therapy. A pilot meta-analysis using literatures with median survival time extractable over a 20-month observation period suggested that PBT, especially CIRT, may be a promising treatment option for PRCC not amenable to curative resection.

## INTRODUCTION

Colorectal cancer is a common disease, with an age-standardized incidence rate of 29 in men and 20 in women per 100 000 person-years in higher Human Development Index countries [1]. The Japanese Cancer Registry estimated that 156 000 cases of colorectal cancers and 52 000 of rectal cancers were newly diagnosed in 2019 [2].

Most local recurrences after colorectal cancer surgery are after rectal cancer, with 0–0.7% of colon cancer and 1.5–4.1% of rectal cancer [3, 4]. The recurrence rate of rectal cancer has decreased with the improvement of surgery by total mesorectal excision and the widespread use of preoperative chemoradiotherapy [5–8]. However, despite improvements in both the neoadjuvant and surgical management of rectal cancer, local recurrence is still an important problem, with documented recurrence rates of 4–8% [9]. Once rectal cancer recurs locally after surgery, it can significantly worsen the health-related quality of life due to intractable pain, bowel obstruction and bleeding [7].

Curative resection of pelvic recurrence of colorectal cancer (PRCC) is the most crucial factor for survival. The treatment strategy for PRCC in the Japanese guidelines for the treatment of colorectal cancer recommends surgical resection only in cases in which complete resection can be inferred to be feasible after detailed evaluation of the extent of the recurrent lesion by diagnostic imaging [3]. When complete resection is not expected, systemic chemotherapy, chemoradiotherapy or radiation therapy are the treatment options. Surgical resection is the primary curative treatment option for PRCC, but most patients are unresectable.

Because a sufficient margin of resection is required for curative resection, total pelvic exenteration (TPE) may be indicated as a surgical technique. However, TPE is a major operation, typically requiring >10 hours of surgery and blood loss of >5000 ml, and is very invasive to the patient. In addition, TPE often results in the loss of many functions and the development of serious post-operative complications such as prolonged wound healing and pelvic infections [10, 11]. Thus, although surgical resection is the first choice of curative treatment for resectable PRCC, many patients are not candidates for it. Therefore, radiation therapy is often the treatment of choice for localized PRCC tumors [12–14].

In general, long-term tumor control with conventional X-ray therapy is difficult in PRCC because of the high probability of radioresistance due to hypoxia [15] and the inability to deliver sufficient doses due to the presence of radiosensitive gastrointestinal (GI) tract and bladder surrounding the recurrent lesions [16]. For this reason, the results of external X-ray radiotherapy (XRT) are still unsatisfactory, and PRCC requires treatment with fewer side effects and better local control (LC). Chemoradiotherapy, stereotactic radiotherapy and particle beam therapy (PBT) have been used to enhance the efficacy of radiotherapy for localized lesions in PRCC [12, 17–19]. Intraoperative radiotherapy [20] or high-dose-rate brachytherapy [21] are also being investigated.

Carbon ion radiation therapy (CIRT) uses charged heavy ion beams with high linear energy transfer properties and has the advantage of utilizing the Bragg peak in terms of dose distribution. Recent publications of CIRT for PRCC from several facilities have shown promising treatment outcomes [22–28].

Proton therapy (PT) is another treatment that utilizes the same physical properties of charged particles as CIRT, with the goal of avoiding high doses to the surrounding normal tissues by improving dose distribution and delivering more effective irradiation to the lesions. In recent years, the efficacy and feasibility of PT for several types of cancers as well as clinical results have been reported [29–33], but there are few reports for PRCC.

In this study, we present the results of a systematic review (SR) of the literature on XRT and PBT and an analysis of PBT registry data for the PRCC.

### Purpose

The purpose of this study is to clarify whether PBT for post-operative PRCC not amenable to resection is superior to XRT and to answer the question, ‘Can PBT be recommended as a treatment for PRCC?’

## MATERIALS AND METHODS

### Registry database

Patients with PRCC treated with particle therapy have been registered in databases, i.e. Proton-Net for PT, J-CROS for CIRT. The study was conducted by the Colorectal Cancer Working Group, a subcommittee of the Particle Therapy Committee of the JASTRO. Ten PT facilities and five heavy particle therapy facilities, including one where both PT and CIRT are available, participated in this multicenter, single-arm, prospective, observational study. Each facility obtained prior approval from an ethics committee and obtained written informed consent from all patients.

Based on the statistical analysis plan, background factors (gender, age, PS (ECOG), history of radiation therapy to the recurrent area (yes/no), chemotherapy to the recurrent tumor (yes/no), site of recurrence, size of recurrent tumor and dose fractionation), treatment details (date of treatment start/end), adverse events (date, type, grade) and prognostic factors (survival status, date and recurrence) were tabulated from the registry database.

### Registry data analysis

Data collected from PRCC patients who underwent PT or CIRT from May 2016 to June 2018 (excluding cases treated with PT for anastomotic recurrence (*n* = 6)) were examined to assess the overall survival (OS), LC rate and treatment-related late toxicity of grade ≥ 3. The duration of follow-up was defined as the period from the start date of PBT to the date of death or the last confirmed date of survival. As a rule, patients were followed every 3 months for the first year after PBT and every 6 months thereafter. OS was defined as time from start of PBT to death regardless of reason. Tumor response to PBT was classified according to Response Evaluation Criteria in Solid Tumors, version 1.1 [34].

OS and LC rates were calculated using the Kaplan–Meier method for the time from the start of PBT to the date of the event or last follow-up visit. Treatment-related late toxicity of Grade ≥ 3, defined as an adverse event occurring 3 months after the completion of PBT, was assessed according to the Common Terminology Criteria for Adverse Events (version 4.0).

### Systematic review

The SR was based on a research protocol describing the aims and methods. The review is reported according to the guideline in the PRISMA statement [35, 36]. The purpose of this search was to determine whether PBT is recommended for post-operative unresectable PRCC.

An expert librarian at the National Institute of Quantum Science and Technology searched articles in the PubMed and Cochrane databases published in English from January 2000 to September 2020. The search strategy of literature was built around a patient, intervention, comparison and outcome flamework [37]. The search strategy included terms such as (colorectal or rectal) and (neoplasms or cancer or tumor) and (proton or carbon ion)/(X-ray or BT or 3D conformal radiotherapy (3D-CRT) or SRT or stereotactic body radiation therapy (SBRT) or radiotherapy). The detail of search terms was as follows:

-Population: (PRCC)

(‘rectal cancer*’ [TIAB] OR ‘colorectal cancer’ [TIAB]) AND

(‘cancer’ [TIAB] ‘cancers’ [TIAB] OR ‘neoplasm’ [TIAB] ‘neoplasms’ [TIAB] OR ‘neoplasms’ [MH] OR ‘tumor’ [TIAB] OR ‘tumors’ [TIAB] OR ‘tumour’ [TIAB] OR ‘carcinoma’ [TIAB] OR ‘carcinomas’ [TIAB]) AND (‘recurren*’ [TIAB])

-Intervention: (PBT)

‘proton therapy’ [TIAB] OR ‘proton radiotherapy’ [TIAB] OR ‘proton beam therapy’ [TIAB] OR ‘proton beam radiotherapy’ [TIAB] OR ‘carbon ion therapy’ [TIAB] OR ‘carbon ion radiotherapy’ [TIAB] OR ‘carbon ion beam therapy’ [TIAB] OR ‘carbon ion beam radiotherapy’ [TIAB] OR ‘heavy ion radiotherapy’ [TIAB] OR ‘heavy ion radiotherapy’ [MH]

-Comparison: (conventional RT)

‘x-irradiation’ [TIAB] OR ‘x-ray irradiation’ [TIAB] OR ‘x-radiation’ [TIAB] OR ‘x-ray radiation’ [TIAB] OR ‘x-ray therapy’ [TIAB] OR ‘x-ray treatment’ [TIAB] OR ‘x-rays’ [MH] OR ‘x-ray therapy’ [MH] OR ‘BT’ [TIAB] OR ‘IMRT’ [TIAB] OR ‘3D-CRT’ [TIAB] OR ‘SRT’ [TIAB] OR ‘SBRT’ [TIAB] OR ‘radiation therapy’ [TIAB] OR ‘radiotherapy’ [TIAB] OR ‘chemoradiotherapy’ [TIAB] OR ‘chemoradiotherapy’ [MH] OR ‘chemoradiation’ [TIAB] OR ‘re-irradiation’ [TIAB].

Two of the authors (S.M. and S.Y.) screened the selected references by titles and abstracts, and full-text copies of all potentially compatible studies were obtained. Then, the published full-text studies that evaluated either XRT or PBT for PRCC were considered for inclusion. Publications of retrospective or prospective clinical trials that reported at least one of such outcomes, e.g. OS, LC rate, median survival time (MST) and rate of Grade ≥ 3 toxicity were eligible, except case studies, abstracts, preclinical studies and review articles. Appropriate literatures through manual searches of articles based on information provided by the SR members were also added. In the second screening, eligibility was assessed independently by eight of the authors (S.M., S.Y., Yu.H., H.Tak., H.Tag., T.K., M.A., Ya.H.), and the final inclusion in the review was based on consensus.

### Meta-analysis of *t*-year survival rate

Studies from the articles selected by SR, that met the criteria of ‘duration of observation was at least 20 months’ and ‘MST could be extracted’, were the subject of a meta-analysis, and the results for the 3-year OS were summarized. Statistical analysis was performed using R software version 4.0.5 and the ‘meta’ package of R software (version 4.18-2) [38].

In cases where the confidence interval (CI) construction methods are clearly different among the articles, the point estimates of *t*-year survival rate *S*(*t*) and the standard error (SE) are unified into the log–log-transformed scale. We obtained estimates and SEs of *S*(*t*) on a unified scale for all studies and performed a random-effects meta-analysis using the asymptotic normality of the estimators. Specifically, this was done using the metagen function of the R meta package. The obtained estimates and CI are backtransformed to obtain the final estimated results.

## RESULTS

### Analysis of registry data

Patient characteristics are shown in Table 1. Between May 2016 and June 2018, 159 PRCC patients treated with PT (*n* = 33; initial primary: rectum/colon = 33/0) or CIRT (*n* = 126; initial primary: rectum/colon = 114/12) were eligible. These patients consisted of 107 men, with a median age of 62 (range: 30–87) years and had a median diameter of 30 mm (range: 8–90) of their tumor size. The median follow-up was 34.0 months (range: 2.1–53.7) for PT and 32.6 months (range: 0.9–56.0) for CIRT.

**Table 1:** Characteristics of the patients with PRCC from the registry dataset

			Patient number *N* (%)	
		Total 159	PT 33 (21)	CIRT 126 (79)
Age				
Median years (range)		62 (30–87)	61 (43–86)	62 (30–87)
Sex				
Male		107 (67)	19 (58)	88 (70)
Female		52 (33)	14 (42)	38 (30)
PS (ECOG)				
0		122 (77)	25 (76)	97 (77)
1		35 (22)	7 (21)	28 (22)
2		2 (1)	1 (3)	1 (1)
Initial primary				
Rectum		147 (92)	33 (100)	114 (90)
Colon		12 (8)	0 (0)	12 (10)
RT history				
Yes		33 (21)	6 (18)	27 (21)
No		126 (79)	27 (82)	99 (79)
Site of recurrence				
Presacral		67 (42)	19 (58)	48 (38)
Pelvic wall		64 (40)	7 (21)	57 (45)
Peritoneal		19 (12)	0 (0)	19 (15)
Other		9 (6)	7 (21)	2 (2)
Tumor size (mm)	Median (range): 30 (8–90)			
Average (median)		32.9 (30)	37.7 (35)	31.6 (29.5)
Dose				
≥70 Gy		150 (94)	29 (88)	121 (96)
<70 Gy		9 (6)	4 (12)	5 (4)

Abbreviations: Gy (RBE), gray (relative biological effectiveness).

With the relative biological effectiveness (RBE) of 1.1 and 3.0 for protons and carbon ions, the dose fractionations for PT were planned to be 60–70 Gy (RBE)/30–35 fr for lesions near the GI tract and 72–75 Gy (RBE)/18–25 fr for those not near the GI tract, and those for CIRT were 57.6 Gy (RBE)/12 fr, 70.4 Gy (RBE)/16 fr or 73.6 Gy (RBE)/16 fr.

**Fig. 1:**
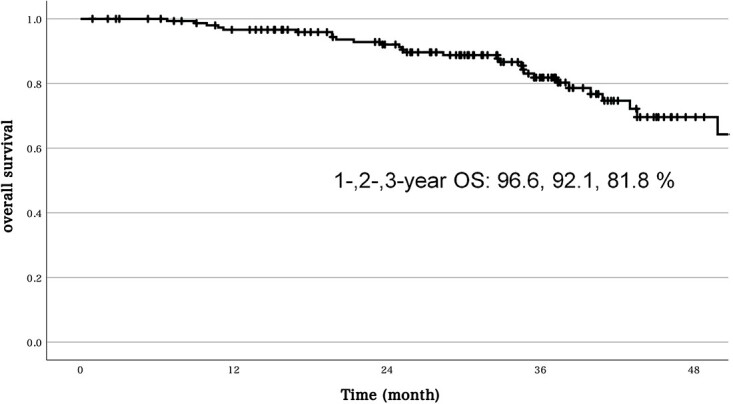
OS of PBT.

**Fig. 2:**
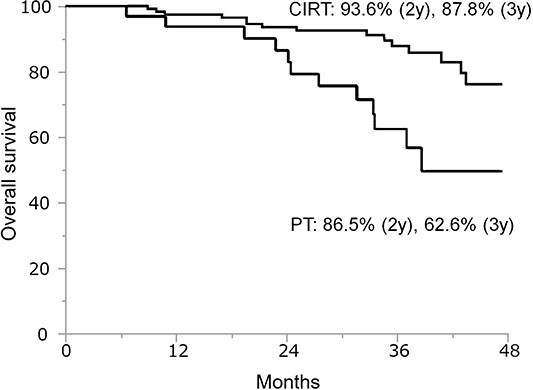
OS of PT and CIRT.

The 1-, 2- and 3-year OS and LC rate of PBT were 96.6, 92.1 and 81.8% and 94.9, 81.3 and 76.4%, respectively (Fig. 1). The 2-/3-year OS of CIRT and PT were 93.6/87.8% and 86.5/62.6%, respectively (Fig. 2). The incidence of Grade 3 or higher late adverse events related to PBT was 5.7%, with Grade 3 neuropathy in three cases; abscess in two cases; rectal ulcer, cystitis and skin ulcer in one case each and Grade 4 rectal perforation in one case.

### Systematic review

**Fig. 3:**
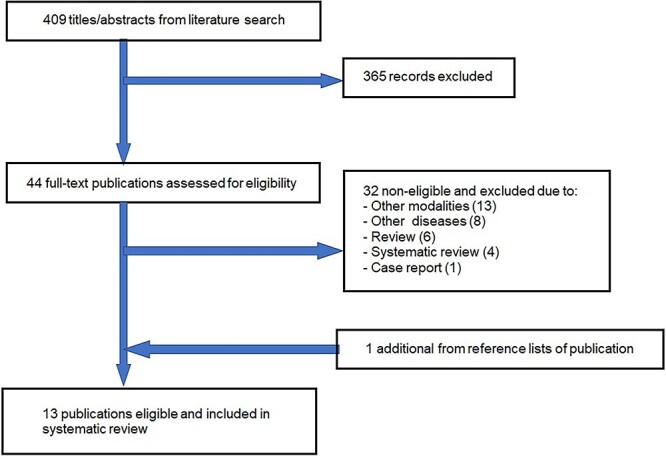
PRISMA flow diagram of the systematic search strategy.

The PRISMA flow diagram of the systematic search for this study is shown in Figure 3. In the primary selection, the systematic search yielded 409 articles. Subsequent secondary screening selected 44 studies, which were further refined to 12 studies. One additional publication was selected using the same procedure from more recent and important literature outside the search period for a total of 13 articles selected for qualitative analysis. Of these 13 studies, 9 were on XRT and the remaining 4 were on PBT. Table 2 summarizes the details of the selected references [12–14, 18, 24, 27, 33, 39–44].

**Table 2:** Summary of the selected articles by SR of the patients with PRCC

Author (year)	Research methods		*N*	Prior-RT(+)	Tumor size (mm): median (range)	Treatment			Follow-up (months)	Outcomes		Toxicity (Grade 3 or more)	
						RT technique	Dose schedule	Combination therapy		LC rate (%)/median (months)	3-year OS (%)	Acute	Late
X-rays													
Hu (2006) [39]^a^	S	P-III	23 (study)	18	N/A	No RT: pelvic exRT + 3D-CRT	40 Gy20 Gy (16–26 Gy)	FOLFOX4 (*n* = 23)	23	N/A	15^b^	GI: 34%, bladder: 4%	0%
			25 (control)	20		Prior RT: 3D-CRT alone	40 Gy(36–46 Gy)	No chemotherapy			N/A	GI: 26%, bladder: 9%	0%
Kim (2008) [18]^a^	S	Retro	23	4	25–80	EBRT + SBRT	45 Gy/2516 Gy (*n* = 5)	Salvage chemotherapy before SBRT (*n* = 23)	31	4y: 74	53^**b**^	1: Gr4 rectal perforation	0%
						SBRT alone	36–51 Gy/3fr (*n* = 18)						
Lee (2011) [40]^a^	S	Retro	22	14	N/A	3D-CRT	57.2 (44.3–74.4)/(1.8–3 Gy/fr)	Chemotherapy	59	5y: 56	52^**b**^		0%
Defoe (2011) [12]	S	Retro	14	14	52.5 (19–110) cm^3^	SBRT (Cyberknife system)	36 Gy/3 fr (*n* = 11), 12 Gy/1 fr (*n* = 1), 16 Gy/1 fr (*n* = 1), 18 Gy/1 fr (*n* = 1)	No	17	2y: 68	59^**b**^	0%	7% (1: pelvic abscess)
Sun (2012) [41]^a^	S	Retro	72	72	N/A	3D-CAHRT	36 Gy/30fr + surgery or 51.6–56.4 Gy	Capecitabine chemotherapy	24	N/A	45	10%	1%
Dagoglu (2015) [13]^a^	S	Retro	18	18	N/A	SBRT (Cyberknife system)	25 Gy/5 fr (24–40 Gy/3–6 fr)	No	38	3y:86	59	1: Gr4 small bowel perforation	1: neuropathy, 1: hydronephrosis
Watanabe (2019) [14]	S	Retro	18	0	49 (15–100)	3D-CRT	59.4 (50–60) Gy	Concurrent chemotherapy (*n* = 18)	54	40.9 mo	54	5: non-hematologic (28%)	0%
												3: hematologic (17%)	0%
Chung (2019) [42]	S	Retro	41	41	33	3D-CRT (*n* = 15)	50 (30–60) Gy	Concurrent chemotherapy (*n* = 21)	42	2y: 29	54	Diarrhea 7%	Fistula 20%, ileus 22%
						IMRT/Cyberknife (*n* = 26)				12.8 mo			
Al-Haidari (2020) [43]^a^	S	Retro	67	67	N/A	3D-CRT (*n* = 63)	45 Gy/1.5 Gy, b.i.d. (*n* = 47)	Concomitant chemotherapy (*n* = 45)	N/A	N/A	32	GI 28%, GU 18%	GI 7%, GU 14%
						IMRT (*n* = 4)	40.8 Gy/1.2 Gy, b.i.d. (*n* = 9)						
PBT													
Hiroshima (2021) [30]^a^	S	Retro	12	0	63 (50–79.2)	PT	50–79.2 Gy (RBE)/20–38 fr	S-1 (6)	36	3y: 80	74	0%	0%
Yamada (2016) [24]	S	Pros Phase I/II	151	N/A	34 (10–140)	CIRT	73.6 Gy (RBE)/16 fr	N/A	42	5y: 88	78	0%	3%
Shinoto (2019) [27]^a^	M	Retro	224	N/A	30 (10–100)	CIRT	70.4, 73.6 Gy (RBE)/16 fr	N/A	62	3y: 93, 5y: 88	73	1%	3%
Shiba (2019) [44]	S	Pros	28	0	44 (16–84)	CIRT	73.6 Gy (RBE)/16 fr	N/A	39	3y: 86	74	0%	7%
The present study													
Registry data	M	Pros	33	6	35 (10–90)	PT	60–70 Gy (RBE)/30–35 fr or 72–75 Gy (RBE)/18–25 fr	No	34		62.6		9%
			126	27	27 (8–76)	CIRT	57.6 Gy (RBE)/12 fr or 70.4 Gy (RBE) or 73.6 Gy (RBE)/16 fr	No	33		87.8		4%
			159	33	30 (8–90)				33	3y: 76.4	81.8	0%	5.7%

Abbreviations: OS = overall survival, S = single site, M = multicenter, P-III = Phase III, Retro = retrospective, Pros = prospective, N/D = no data, CAHRT = conformal accelerated hyperfractionation radiotherapy, N/A = not available, GU = genitourinary, EBRT = external beam radiotherapy, IMRT = intensity-modulated radiotherapy, b.i.d. = bis in die.

^a^Adopted for meta-analysis.

^**b**^Manual acquisition from figure.

All XRT articles were reported by a single-site study, including a Phase III study of 3D-CRT with or without chemotherapy for unresectable recurrent rectal cancer. Of the nine XRT studies, six were conducted using 3D-CRT with conventional fractionation or accelerated hyper-fractionation, and three were conducted using stereotactic body radiotherapy (SBRT) with three to six fractions. Of the four PBT studies, three were conducted by CIRT and one by PT. One of the CIRT studies was conducted as a multicenter study.

In the XRT study series, 3-year OS was reported in the range of 15–59%, but LC rates were not reported in a comparable manner. The PBT study series reported a similar 3-year survival rate of 71–78% using particle therapy alone and a unified dose fractionation regimen. The incidence of Grade 3 or higher toxicity in each study was also listed in Table 2, separately for the acute and late phase when extractable. In the XRT series, incidences of acute GI toxicity ranged from 0 to 34%, while in the PBT series they were <0%. Severe acute adverse events in XRT patients were predominantly observed in the treatment group, which included patients who were re-irradiated or who received concurrent chemotherapy. The frequency of late GI toxicity was similar for XRT and PBT, 0–7%. However, a high incidence of Grade 3 or higher fistula formation was reported in the group of patients treated with concurrent chemotherapy and re-irradiation, who received a median dose of 50 Gy of XRT [43].

### Meta-analysis

From the four articles on PBT for PRCC, one study on PT and one study on CIRT were selected for meta-analysis; the selection of the CIRT literature took into account the overlap in study subjects based on the Heavy Ion Therapy Multicenter Database (Table 2).

**Fig. 4:**
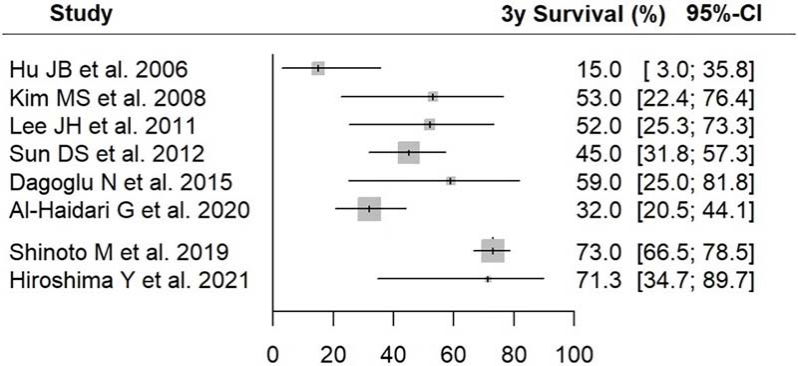
Estimates of 3-year survival and 95% confidential interval.

The 3-year OS and CIs calculated from the six XRT and two PBT articles using the random-effect model were 39.7% [27.4–51.8] and 72.9% [66.5–78.3], respectively, and those obtained from registry data were 81.8% [74.5–89.1]. The 3-year OS estimates and 95% CIs for each of the eight articles calculated during the meta-analysis process are summarized in Figure 4.

## DISCUSSION

In Japan, XRT for PRCC is covered by insurance under the National Health Insurance System. However, prior to March 2022, PBT for PRCC was a medical service that was not covered by public insurance, whose effectiveness should be evaluated as an advanced medical treatment. Recognizing the importance of properly comparing the efficacy of these treatments for PRCC, the Japanese Society for Radiation Oncology (JASTRO) has enrolled and followed all patients undergoing PBT with the goal of obtaining public insurance coverage. The Colorectal Cancer Working Group of the JASTRO Particle Therapy Committee continued to examine prospectively collected registry data and systematically reviewed clinical outcomes and patient prognoses for each treatment modality obtained from literature data.

A multicenter prospective registry of patients undergoing PBT in Japan has been running since May 2016, and the studies of PT [45] and CIRT [46] have been registered in the University Hospital Medical Information Network Center.

Although R0 resection is considered as the most acceptable prognostic factor in the treatment strategy for PRCC in the Japanese colorectal cancer treatment guidelines [3], the R0 resection rate is not always high, and there is wide variation among reports. One of the reasons for this is that curative resection for local recurrence requires technically extraordinarily difficult procedures such as TPE with sacrectomy. Therefore, resection is considered only when R0 resection is deemed feasible. Preoperative radiotherapy, chemoradiotherapy and intraoperative irradiation have been used to improve the R0 resection rate of PRCC. Superior outcomes of CIRT for PRCC have been published, and CIRT may be the treatment of choice for patients who cannot be expected to undergo R0 resection or who refuse surgery [24, 27].

Of the patients with PRCC treated with PBT from the registry dataset, 12 patients of CIRT had colon cancer as the initial primary disease, however, there was no colon cancer case as initial primary in the PT registry. Therefore, results for PT patients in this study were limited to cases of local recurrence of rectal cancer.

Registry data showed that CIRT was superior than PT in survival. For comparisons of the anti-tumor effect, the BED_10_ (assuming an *α*/*β* value of 10) values converted from the prescribed doses for CIRT were 101.4 Gy_10_ and 107.5 Gy_10_ for 70.4 Gy (RBE)/16 fr and 73.6 Gy (RBE)/16 fr, respectively. On the other hand, similarly converted BED_10_ for PT was smaller than that of CIRT, with a median of 93.6 (range: 60.0–103.9) Gy_10_ for an actual TD/fraction with a median of 72 Gy (RBE)/24 fr. This could be one of the reasons for the superior 3-year OS of CIRT over PT in the analysis of the PBT registry database. However, there are other differences, such as more presacral lesions and larger median tumor diameter in the PT group, which may also cause the difference in treatment results between CIRT and PT in the registry data [27]. On the other hand, the SE value in the distribution of OS estimates calculated from the SR literature is much larger for PT than for CIRT, reflecting the lack of treatment experience, and the application of PT to PRCC may require concurrent chemotherapy as well as XRT.

Our SR revealed that there was one Phase III study in the XRT literature, one Phase I/II study and one prospective observational study each in the CIRT literature, while the other 10 studies were all retrospective observational studies. Most of the studies in the XRT literature were conducted in combination with chemotherapy. The PBT publications were as the results of advanced medical treatment from Japanese facilities. The PBT papers reported multicenter results with minor variation, with 3-year survival rates of 73–78% and LC rates of 80–93% because the dose-fractionation methods and other factors were standardized.

Recently, Venkatesulu *et al*. reported an SR study of CIRT for recurrent rectal cancer. They found promising results with CIRT for this difficult-to-treat condition and noted the need to compare CIRT with PT or XRT in further studies to establish clinical treatment recommendations [47]. No randomized trials directly comparing XRT and PBT as a definitive treatment for PRCC was found in the current SR literature. In this study, we attempted a meta-analysis by calculating the estimated 3-year OS and its CI from the XRT and PBT study data obtained in SR. In comparison with the XRT SR literature with PBT SR literature, the upper CI of the XRT SR literature is lower than the lower CI of PBT for 3-year survival rate. However, except for the fact that the target of treatment in the collected articles was recurrent colorectal cancer, the data were obtained from patients with widely different backgrounds in terms of factors, such as previous irradiation and concurrent chemotherapy, and it is difficult to give a clear meaning to the comparison between the integrated data obtained from the model calculation based on these estimates. However, the plausibility of the estimated 3-year survival rate of CIRT is reflected in the very small standard deviation compared to PT, which has a numerically close 3-year survival rate as seen in Figure 4.

We found no incidence of treatment-related acute toxicity above Grade 3, which was also the case in the PBT SR extraction literature, while the XRT study showed a higher frequency of acute adverse events, especially when chemotherapy was used in combination. The registry data analysis also showed Grade 4 proctitis in the late phase. On the other hand, the XRT study reports a high frequency of late GI tract toxicity in patients with recurrent disease who were treated with irradiation around the time of their first surgery; it is possible that the distance between the recurrent tumor and the GI tract correlates with the frequency of late toxicity with PBT, but the small number of cases requires long-term observation to confirm this.

In conclusion, PBT for PRCC may be a treatment option aimed at LC. In particular, CIRT for localized recurrent lesions is likely to be recommended as a promising treatment option for local cure.

## Data Availability

There are restrictions on the release of registration data to the outside community.
